# Factors Influencing the Accuracy of Freehand Implant Placement: A Prospective Clinical Study

**DOI:** 10.3390/dj9050054

**Published:** 2021-05-10

**Authors:** Sigmar Schnutenhaus, Marie Wagner, Cornelia Edelmann, Ralph G. Luthardt, Heike Rudolph

**Affiliations:** 1 Center of Dentistry, Dr. Schnutenhaus MVZ GmbH, 78247 Hilzingen, Germany; edelmann@schnutenhaus.de; 2 Department of Prosthetic Dentistry, Center of Dentistry, Ulm University, 89081 Ulm, Germany; marie-1.wagnr@uni-ulm.de (M.W.); ralph.luthardt@uniklinik-ulm.de (R.G.L.); heike.rudolph@uniklinik-ulm.de (H.R.)

**Keywords:** accuracy, computer-assisted surgery, computer-guided surgery, cone-beam computed tomography, dental implant, freehand

## Abstract

(1) Background: The objective of implant prosthetic restoration is to ensure the best possible rehabilitation of function and esthetics. Optimal positioning of the implant with regard to the bone availability, surrounding soft tissue, and prosthetic sustainability should be strived for during implant placement. The factors influencing freehand implant placement and the accuracy achieved with this procedure are investigated in this prospective clinical study. (2) Methods: Implants were placed in the single-tooth edentulous sites of the premolar and molar areas in 52 patients. Three-dimensional (3D)-planning was performed virtually prior to the freehand implant operation, and the desired position of the implant was provided to the surgeon. (3) Results: The deviations between the planned and the actually achieved position with freehand implant placement showed the following mean values and standard deviations: angle 8.7 ± 4.8°, 3D deviation at the implant shoulder 1.62 ± 0.87 mm, mesiodistal deviation 0.87 ± 0.75 mm, buccolingual deviation 0.70 ± 0.66 mm, and apiocoronal deviation 0.95 ± 0.61 mm. The type of jaw had a significant influence on accuracy. Major deviations were observed in the lower jaw. Furthermore, the timing of implant placement influenced the mesiodistal deviation and angular deviation; (4) Conclusions: Freehand implant placement demonstrated a higher level of deviation between the planned and actually achieved implant positions. In particular, the ranges showed a large spread. From a prosthetic point of view, there may be complications during the restoration of the prosthetic crown if the implant exit point is not optimally located or if the implants show a high angular deviation.

## 1. Introduction

The loss of teeth leads to restrictions in chewing function and often in esthetics. Implant prosthetic restorations are intended to remedy these limitations. To achieve this, the implants must be placed in the correct position to ensure a durable and predictable prosthetic restoration. With three-dimensional (3D) prosthetic-based planning, the available bone can be optimally utilized. Such planning can be implemented using computer-assisted surgical procedures. Template-guided placement has been established as a static procedure, whereas real-time navigation is a dynamic process [1]. Freehand implant placement is still used in the conventional method. Implantation must avoid damage to adjacent anatomical structures in addition to achieving an optimal implant position according to prosthetic principles. Guided implant placement can be helpful in complex cases. Studies have shown that guided procedures can improve clinical outcomes, especially in the aesthetic zone [2]. If immediate restorations are planned and will be placed during implant placement, this is difficult to perform with the freehand method. Such restorations can be implemented predictably with a digital protocol [3]. The surgeon’s experience and the anatomical condition have been assessed as factors influencing the accuracy of a freehand procedure [4].

Implant transfer accuracy and its influencing factors in conventional and computer-assisted implant placement methods have already been the subject of a large number of published studies and reviews. Comparisons of template-guided implant placements with conventional freehand implant placements showed significantly more precise results in favor of the guided procedures [5,6,7,8]. Tahmaseb et al. examined 20 clinical studies with regard to the accuracy of statically guided implants. As a result of the meta-analysis, deviations at the shoulder of the implant were calculated to be 1.2 mm at the mean and 1.4 mm at the apex. The angular deviation of the implant axis was 3.5° on average [9]. In a randomized clinical study by Varga et al., the deviations resulting from freehand implant placements and the various implant placements with the aid of drilling templates were compared. They found an average angular deviation of 7.03° in a freehand procedure with a range of 0.7–21.3°. For sleeve-guided template systems, a mean angular deviation of 3.04° and a range of 0.4–6.3° was observed [10]. Large deviations between the prosthetic-based planned implant position and the actual position achieved can have a direct influence on the prosthetic care ability of the implants, particularly, in the case of screw-retained single implants as used in this study. Therefore, the deviation at the implant shoulder and angle deviation are decisive parameters from a prosthetic point of view. Consideration of the deviations at the apex, on the other hand, indicates anatomical situations and thus, surgical risk factors.

The aim of this prospective clinical study was to determine the accuracy of freehand implant placement after 3D planning performed by the surgeon. In addition, the factors that affect the accuracy of freehand implant placement were investigated. The hypothesis of this study was that both the jaw and the location of the implant influence the accuracy of implant placement. Further, we hypothesized that accuracy shows no dependence on the time interval of implant placement after tooth extraction.

## 2. Materials and Methods

### 2.1. Patients

A total of 52 patients were included in this prospective clinical study. Patients were recruited following registration of the study with the WHO (DRKS-ID: DRKS00009628) and approval by the responsible ethics committee (Ethics Committee of Ulm University, Application No.: 41/14, approved on 6 May 2014). Implants were placed during the period from February 2016 to April 2020. Patients’ data were collected in the practice of Dr. SiS in Hilzingen (cooperating partner of the Clinic for Dental Prosthetics, Ulm University Clinic). In this study, only single-tooth edentulous sites in the premolar and molar areas were included. The timing of implant placement included early implant placement (Type 3: 3–6 months post-extraction) and late implant placement (Type 4: >6 months post-extraction) as well [11].

### 2.2. Inclusion Criteria

To participate in the study, patients had to meet the following criteria:

Inclusion criteria:

- Submission of written informed consent;
- Restoration of a missing tooth using an implant;
- Restoration of single-tooth edentulous sites in the premolar or molar region;
- Sufficient existing bone. The implant had to be surrounded by 1.5 mm of bone on all sides after evaluation of the CBCT;
- Planned prosthetic restoration with occlusal screw-retained hybrid-abutment crowns;
- Healing of the extraction socket for at least 3 months.

Exclusion criteria:

- Patients under 18 years or without any legal capacity;
- Use of an implant placement template is not possible (restricted mouth opening);
- Heavy smoker (>10 cigarettes/day);
- known systematic conditions to alter bone metabolism or affect wound healing (e.g., bisphosphonates);
- Pregnant women;
- Alcohol and/or drug abuse;
- Patients with infectious diseases such as hepatitis, HIV, or AIDS;
- Patients with uncontrolled severe diabetes mellitus. The long-term glucose parameter HbA1c value was required to be below 6.7%.

### 2.3. Planning

After obtaining written consent from the patients, cone-beam computed tomography (CBCT) (Gendex CB500, Gendex Dental Systems, Des Plaines, IL, USA) was performed. The CBCT scans were performed in a slightly open mouth position to display the rows of teeth without overlap. The image was acquired with a constant 0.2 voxel resolution. For implant planning, an alginate impression of the respective jaw was made to fabricate a diagnostic plaster model followed by a prosthetic wax-up which was optically scanned (3Shape Scanner D 700, 3Shape A/S, Copenhagen, Denmark) for each patient. The 3D implant planning was carried out using the implant planning software, SMOP (Swiss-Meda Operations-Planning, Swiss-meda, Zurich, Switzerland). In this software, the CBCT data were superimposed with the data records of the planning and the digitized initial situation. This superimposition was performed semi-automatically in the program. The optimum implant position was then planned virtually. The bone volume, the soft tissue situation, and the prosthetic planning objective were all taken into account. All planning steps and subsequent implant placement were performed by the same experienced practitioner (SiS). 

### 2.4. Implant Placement

After planning, implant placement was performed using a freehand procedure. All surgical procedures were performed under local anesthesia. After elevating a minimal mucoperiosteal flap, the implant bed was prepared according to the manufacturer’s protocol. Transgingival healing implants (iSy, Camlog, Wimsheim, Germany) were used. During implant placement, the planning was displayed on a monitor, and the surgeon was briefed to position the implant according to this plan as much as possible. 

### 2.5. Registration of the Implant Position

All implants were restored with screw-retained hybrid abutment crowns. For the prosthetic restoration, the clinical situation was recorded 6–12 weeks after implant placement with the help of an individual spoon, using a transfer cap that was attached to the insertion post and additional silicone impression material (imprint quick, 3M Espe, Seefeld, Germany). All impressions were made by the practitioner. After disinfection, the impression was poured to fabricate a plaster model by a dental technician. The impression post was then supplemented with a screwed-on analogous implant, and the impression was digitized (3Shape Scanner D 700, 3Shape A/S, Copenhagen, Denmark).

### 2.6. Alignment of Datasets

Overlay datasets were created using the Geomagic Studio program (version 9, Geomagic, NC, USA). All data were consecutively analyzed in terms of location and time by an investigator, regardless of their generation. Data records of the digitized implant impressions were exported as an interface file in STL format. The latter represents the clinically achieved implant position. The digitized gypsum planning model, which was aligned to the CBCT data in the planning program (SMOP), as well as the truncated cones representing the planned implant position, were exported from the planning program (SMOP) and served as the reference dataset. 

Datasets were reduced to a defined structure, the unchangeable hard tooth substance, in order to exclude errors due to soft tissue changes or deviating implant positions. The alignment quality was assessed on the basis of the Root Mean Square value (RMS, the root of the mean value of the squares of all deviations). 

For the planned analysis of the distance and angle deviations, the use of auxiliary constructs was necessary, which reflected the exact planned position of the implant and the clinically achieved implant position. It was created with the help of the Surfacer 10.6 program (Imageware, Ann Arbor, MI, USA) using simple geometric shapes. The auxiliary constructs were adapted to the respective implant lengths and diameters and then loaded into the Geomagic Studio program for assignment. In this way, it could be ensured that the axis endpoints and axis deviation of the implant positions could be determined in a standardized manner for further analysis. This methodology has already been extensively used and described by Schnutenhaus et al. [12].

The assigned auxiliary constructs, which reproduced key data of the 3D information of the planned and clinically achieved implant position, were loaded into the Surfacer 10.6 Imageware program for further analysis. 

### 2.7. Analysis of the Implant Position

**Figure 1:**
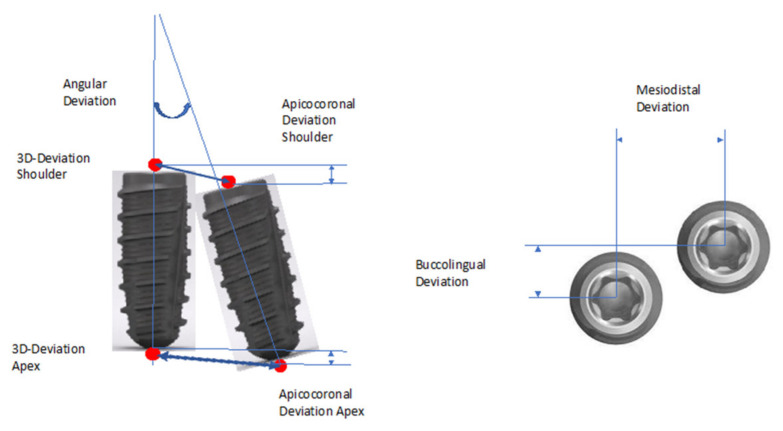
Representation of the measuring distances and the angular deviation between the planned and actual achieved implant position.

The metric analysis included the following measurements (Figure 1). The deviations between the planned and the actual implant position were measured. Length measurements were taken from the center of the implant at the shoulder and at the apex.

- Three-dimensional (3D) deviation.
- Two-dimensional deviations in apicocoronal direction (height deviation) and mesiodistal and buccolingual direction.

Axis deviation: The measurement method was based on the principle of Tahmaseb et al. [13] to enable better comparability with current and future studies.

### 2.8. Statistical Analysis

The mean values, standard errors, and 95% confidence intervals were given for the variables. After testing for normal distribution, statistical testing according to the Kruskal-Wallis test was performed for values that were not normally distributed. Given the exploratory nature of this study, all statistical results must be interpreted as hypothesis-generating and not confirmatory. All statistical tests had a significance level of α = 0.05 (two-sided test). No adjustments were made for the multiple tests. Statistical analysis was performed using IBM SPSS Statistics Version 26.0 (IBM Corp. Released 2019, Armonk, NY, USA).

## 3. Results

### Description of the Study Population

Of the 52 patients, 34 were women and 18 were men. The average age was 50.3 years (range, 22–75 years). The distribution of tooth regions is shown in Table 1. 

The lengths and diameters of the placed implants are listed in Table 2.

The assignment and overlay of the data records were performed using clearly identifiable hard tooth substances. When assigning the interface data records from the planned implant position and the clinically achieved implant position, the mean RMS error was 85.6 µm (SD 25.4).

The evaluation data of all 52 patients are shown in Table 3.

The evaluation of all implants showed a mean angular deviation of 8.7° and a 3D deviation of 1.62 mm at the shoulder. The distribution of the directional deviations at the prosthetically relevant exit point showed that 59.6% of the implants were placed considerably distal from the planned position. In the buccolingual direction, 53.8% of the implants were placed further lingual, and in the apicocoronal direction, 55.8% of the implants were placed higher than planned.

Data were analyzed with regard to implant placement regions. The results for the upper and lower jaws (Table 4) were compared. Another analysis examined the differences in the premolar and molar regions (Table 5). An evaluation of the sides (right/left) was also performed (Table 6). Another factor investigated was whether the timing of implant placement (early (Type 3)/late (Type 4)) had an influence on the variables (Table 7). 

There were no significant differences in the premolar vs. molar and right side vs. left side factors. However, the jaws had a significant impact on the accuracy of the mesiodistal deviation. A higher number of deviations was observed in the lower jaw.

The timing of implant placement also had a significant influence on accuracy. Early implant placements showed higher deviations in both the angle and mesiodistal deviation than were found with late implant placement.

## 4. Discussion

An in vitro study by Jorba-Garcia et al. demonstrated that the freehand method was significantly less accurate compared to a dynamically guided method [14]. This particularly affected the angular deviation, which averaged 1.6° for dynamic navigation and 9.7° for freehand navigation. Similar values were found in the in vitro study by Chen et al., where the angular deviation at the implant shoulder was 4.45° ± 1.97° with the guided procedure. In contrast, the angular deviation in the freehand procedure was 9.26° ± 3.62° [15]. These values were significantly different. The limits of freehand implantation are clearly marked from these studies. This is an indication that computer-assisted procedures should be preferred for specific indications.

In a cadaver study using the freehand method, deviations at the implant shoulder averaged 1.43 mm (range: 0.65–2.31 mm), 2.20 mm at the apex (range: 1.01–4.02 mm) with an angular deviation of 6.78° (range: 3.08–14.98°) [16]. Another cadaver investigation was conducted by Noharet et al. [17]. A comparison of the in vitro studies with the clinical studies shows that an “implant surgery” can be carried out much more precisely on a model or on the cadaver.

In a clinical study by Vercruyssen et al., significantly higher values were found [8]. Various template-guided methods were examined in comparison with the freehand method. With the freehand method, there were deviations on the implant shoulder averaging 2.77 mm (range: 0.33–8.34 mm), 2.91 mm at the apex (range 0.53–7.37 mm), and an angular deviation of 9.92° (range: 1.45–27.76°). Varga et al., in their clinical study of freehand implant bed preparation, found deviations at the implant shoulder averaging 1.82 mm (range: 0.56–5.38 mm), 2.43 mm at the apex (range 0.54–4.83 mm), and an angular deviation of 7.03° (range: 0.71–21.30°) [10]. Further clinical studies constantly showed significant differences in all parameters of accuracy and a large range between the minimum and maximum values [6,10,18,19]. In particular, a high number of deviations in the angular dimensions have been found in clinical studies. For example, Aydemir and Arisan found a mean deviation of 10.04° with a range of 2.19–20.42° when using the freehand method [20]. All studies show sufficiently accurate values in the mean of the deviations. However, they all confirm a high degree of scatter in the maximum values. These maximum values are worth considering from both a surgical and a prosthetic point of view. Therefore, a determination of the influencing factors appears helpful. In the present study, tooth-delimited single-tooth edentulous sites were restored with implants. Smitkarn et al. examined the accuracy of implant placement in single-tooth edentulous sites. With respect to angular deviation, they achieved a mean value of 6.9° with a range of 0.5–16.9°. The values for the lateral and 3D deviation were within the range of the present study (mesiodistal mean value: 0.6 mm (range 0.03–1.67 mm), buccolingual mean value: 0.5 mm (range 0.00–2.14 mm), apicocoronal mean value 1.0 mm (range 0.03–3.95 mm), and 3D deviation mean value of 1.5 mm (range 0.39–4.03 mm)) [21]. In contrast to the studies mentioned above, Efstathiou et al. showed only minor axial deviations in freehand implantation [22]. However, they performed their measurements two-dimensionally after evaluation of panoramic radiographs. On the one hand, this could be an indication of the influence of the measurement procedure. On the other hand, this could also be a confirmation that the surgeons’ skills in spatially correct implant positioning are the decisive influencing factor.

The values observed in this study, as well as the present study, elucidate that the ranges, in particular, have a decisive influence on prosthetic sustainability in supposedly simple situations. The patients who exhibited high deviation values in the present study were subsequently traced back. It was found that these patients, in particular, had restricted mouth openings or demonstrated little cooperation during the operation. This was observed in addition to confirming the hypothesis that accuracy is dependent on the anatomical situation and the region of implantation. The accuracy that can be achieved with freehand implant placement depends significantly on the surgeon’s experience. In an in vitro study, the freehand method performed by an experienced implant surgeon showed angular deviations with an average of 6.69° compared to an inexperienced surgeon (12.66°) [14]. Even with the use of a drilling template, the experience of a surgeon had a significant influence on the outcome [23]. 

Comparing the present study with template-guided implant placement minimizes the surgeon’s experience and individual skills as influencing factors. In a study of 122 implants, the mean values of the deviations at the shoulder were 1.2 mm (95% CI 0.1–3.8 mm), 1.8 mm at the apex (95% CI 0.35–0.1 mm), and an angular deviation of 4.8° (95% CI 0.02–14.6°) [24]. In a modified template design with two sleeve guides, the mean values at the shoulder were 0.52 mm (95% CI 0.37–0.67 mm), 0.82 mm at the apex (0.56–1.08 mm) and the angular deviation was 2.8° (2.18–3.51°) [25].

In a study by Vercruyssen et al., there was no difference in patient satisfaction and postoperative pain following implant placement when the free hand and template-guided procedures were compared [26]. However, with regard to postoperative pain and swelling, Pozzi et al. found significantly higher values with the freehand method than with template-guided implant placement [27]. It is known that the presence of an adjacent tooth and the quadrant and the location of the implant site influences the direction and angulation deviation of the implant position [4,28]. This is partially confirmed in the present study. In addition, the influence of the implantation time was found here as a further influencing factor. One explanation could be the degree of mineralization of the newly formed bone and thus its density.

In the present study, only implantations without additional bone augmentation were included. Under the aspect that the implant position can be modified by the possibility of bone augmentation, new influencing factors may arise [29].

Sun et al. showed that the location of implantation had a significant influence on the accuracy of the 3D deviation and angular deviation [19]. This enabled the achievement of higher levels of accuracy in the upper jaw and anterior regions. Implant location exhibited a significant influence on accuracy in an in vitro study by Abduo and Lau. In a comparison between an implant placed in the central incisor area and the first molar area, a lower level of accuracy was measured in molar area for horizontal deviation at the implant shoulder and at the apex of the distal implant [30]. In our study, the hypothesis that the location of the implant has an influence on accuracy could not be confirmed. Arisan et al. presented the limits of freehand implant placement when restoring edentulous jaws. When assessing the implant position using the freehand method, 54.65% of the implants were classified as inadequately positioned in comparison to a mucosa-supported drilling template, where this value was reduced to 15.78% [31].

The ultimate influence of the implant placement procedure, freehand or computer-assisted implant placement, has hardly been worked out so far. In a case series, Baldi et al. could not find any differences in the restoration of maxillary anterior teeth with these two different procedures [32]. They see the advantages of the digital approach in the fact that a consistent prosthetic workflow can be maintained, for example with long-term provisional restorations fabricated in advance. 

It has been demonstrated that successful implantation does not depend on the surgical procedure [27]. After an observation period of 3 years, Bernard et al. found no significant differences between template-guided and freehand placement of implants [33]. Pozzi et al. could not determine any differences between freehand and template-guided implant placements in a one-year follow-up study. Among other variables, they examined the implant survival rate, complications, and peri-implant bone loss [27]. The meta-analysis by Yogui and coworkers confirms the statement that there are no significant differences in marginal bone loss, mechanical and biological complications, and implant survival rate between these two methods [34]. However, it could be determined that the conventional surgical method showed significantly higher levels of discomfort in patients, such as pain and swelling than in computer-assisted procedures [35]. This is also reflected in the patient-related outcome measures. The majority of patients prefer a computer-assisted approach as found by Sancho-Puchades et al. in their randomized clinical trial [36].

## 5. Conclusions

Freehand implant placement is a proven procedure. Studies have shown that implant survival rates do not depend on the surgical method. In the present study, it was shown that the optimal implant position could not be adequately achieved in every case using the freehand method. If there is a significant discrepancy between the achieved position and the optimal planned implant position in terms of angular deviation and implant emergence point, a predictable restoration is questionable from a prosthetic point of view. Within the limitations of the study, it has been shown that larger deviations are possible in difficult anatomical conditions or less accessibility to the surgical site. The use of computer-assisted surgical procedures can, therefore, be an alternative to minimize such deviations and can be recommended from the perspective of prosthetic rehabilitation.

## Figures and Tables

**Table 1:** Distribution of the implants according to regions and tooth groups (premolars or molars).

	Right (N = 22)	Left (N = 30)
Upper jaw (N = 20)	Premolars: 6 Molars: 1	Premolars: 8 Molars: 5
Lower jaw (N = 32)	Premolars: 3 Molars: 12	Premolars: 7 Molars: 10

**Table 2:** Distribution of implant lengths and diameters (mm).

Lengths/Diameters	3.8	4.4	5.0
9	5	9	6
11	11	11	8
13	1	1	0

**Table 3:** Angular deviation (°) and deviations at the shoulder and at the apex (mm) in the total group with 52 implants.

		Mean	Standard Deviation	Minimum	Maximum
Angle	Deviation	8.70	4.8	1.8	22.3
Shoulder	3D deviation	1.62	0.87	0.13	3.57
	Mesiodistal deviation	0.87	0.75	0.00	3.13
	Buccolingual deviation	0.70	0.66	0.00	2.96
	Apicocoronal deviation	0.95	0.61	0.00	2.32
Apex	3D deviation	2.68	1.52	0.48	7.12
	Mesiodistal deviation	1.93	1.51	0.09	6.51
	Buccolingual deviation	1.10	1.03	0.15	4.36
	Apicocoronal deviation	1.05	0.72	0.41	2.82

**Table 4:** Deviations between the planned and achieved implant position according to the jaws. Upper jaw (N = 20) lower jaw (N = 32). Angle variance (°). Linear deviation (mm). Kruskal–Wallis test with a significance level of *p* < 0.05.

		Upper Jaw		Lower Jaw		
		Mean	SE (95% CI)	Mean	SE (95% CI)	Sig. Level
Angle	Deviation	6.98	0.89 (5.11–8.84)	9.77	0.90 (7.94–11.60)	0.050
Shoulder	Mesiodistal deviation	0.54	0.13 (0.27–0.82)	1.07	0.14 (0.80–1.35)	0.011 *
	Buccolingual Deviation	0.71	0.14 (0.41–1.01)	0.69	0.12 (0.44–0.94)	0.742
	Apicocoronal Deviation	0.97	0.14 (0.67–1.27)	0.94	0.10 (0.72–1.15)	0.903
Apex	Mesiodistal deviation	1.36	0.27 (0.81–1.92)	2.28	0.28 (1.70–2.85)	0.038 *
	Buccolingual deviation	1.27	0.22 (0.80–1.74)	0.99	0.19 (0.61–1.37)	0.140
	Apicocoronal deviation	1.01	0.16 (0.67–1.34)	1.08	0.13 (0.82–1.35)	0.645

* indicates statistical significance.

**Table 5:** Deviations between the planned and achieved implant position depending on the tooth region. Premolar (N = 24) Molar (N = 28). Angle variance (°). Linear deviation (mm). Kruskal–Wallis test with a significance level of *p* < 0.05.

		Premolar		Molar		
		Mean	SE (95% CI)	Mean	SE (95% CI)	Sig. Level
Angle	Deviation	7.43	0.76 (5.86–8.99)	9.78	1.04 (7.66–11.91)	0.163
Shoulder	Mesiodistal deviation	0.68	0.12 (0.44–0.92)	1.03	0.16 (0.70–1.36)	0.169
	Buccolingual deviation	0.70	0.13 (0.43–0.96)	0.70	0.13 (0.43–0.97)	0.963
	Apicocoronal deviation	1.02	0.12 (0.77–1.28)	0.89	0.12 (0.65–1.12)	0.354
Apex	Mesiodistal deviation	1.47	0.25 (0.97–1.98)	2.31	0.31 (1.67–2.95)	0.077
	Buccolingual deviation	1.30	0.21 (0.87–1.73)	0.93	0.19 (0.53–1.32)	0.100
	Apicocoronal deviation	1.04	0.13 (0.76–1.31)	1.07	0.15 (0.76–1.37)	0.833

**Table 6:** Deviations between the planned and achieved implant position depending on the sides. Right side (N = 24) Left side (N = 28). Angle variance (°). Linear deviation (mm). Kruskal–Wallis test with a significance level of *p* < 0.05.

		Right Side		Left Side		
		Mean	SE (95% CI)	Mean	SE (95% CI)	Sig. Level
Angle	Deviation	8.78	1.08 (6.54–11.02)	8.62	0.85 (6.87–10.37)	0.854
Shoulder	Mesiodistal deviation	0.77	0.15 (0.45–1.09)	0.95	0.14 (0.37–1.24)	0.364
	Buccolingual deviation	0.68	0.12 (0.44–0.92)	0.71	0.14 (0.43–1.00)	0.755
	Apicocoronal deviation	1.04	0.10 (0.83–1.25)	0.87	0.13 (0.61–1.14)	0.088
Apex	Mesiodistal deviation	1.79	0.32 (1.14–2.45)	2.04	0.28 (1.46–2.62)	0.474
	Buccolingual deviation	1.19	0.25 (0.67–1.71)	1.02	0.16 (0.69–1.34)	0.941
	Apicocoronal deviation	1.12	0.12 (0.88–1.37)	0.99	0.16 (0.67–1.31)	0.169

**Table 7:** Deviations between the planned and achieved implant position depending on the timing of implant placement. Early implantation (N = 21) Late implantation (N = 31). Angle variance (°). Linear deviation (mm). Kruskal–Wallis test with a significance level of *p* < 0.05.

		Early Implant Placement		Late Implant Placement		
		Mean	SE (95% CI)	Mean	SE (95% CI)	Sig. Level
Angle	Deviation	11.03	1.05 (8.85–13.22)	7.11	0.76 (5.55–8.67)	0.005 *
Shoulder	Mesiodistal deviation	1.06	0.15 (0.76–1.36)	0.74	0.14 (0.45–1.03)	0.050
	Buccolingual deviation	0.86	0.18 (0.49–1.24)	0.59	0.09 (0.40–1.24)	0.275
	Apicocoronal Deviation	1.08	0.14 (0.79–1.37)	0.86	1.04 (0.65–1.07)	0.182
Apex	Mesiodistal deviation	2.62	0.30 (1.99–3.24)	1.46	0.26 (0.63–1.98)	0.003 *
	Buccolingual deviation	1.41	0.29 (0.80–2.01)	0.89	0.13 (0.62–1.16)	0.309
	Apicocoronal deviation	1.27	0.18 (090–1.64)	0.91	0.11 (0.68–1.14)	0.081

* indicates statistical significance.

## Data Availability

The complete documentation of all patients enrolled in this study belongs to the authors and is available only upon reasonable request.
